# Dynamics of IDH1 Mutation and Protein Expression Between Primary and Recurrent Gliomas and Their Prognostic Significance

**DOI:** 10.1155/humu/7543241

**Published:** 2026-10-27

**Authors:** Wei Yu, Siyu Guo, Beibei Liu, Qichun Wei

**Affiliations:** ^1^ Department of Radiation Oncology Second Affiliated Hospital Zhejiang University School of Medicine Hangzhou Zhejiang China zju.edu.cn; ^2^ Cancer Institute (Key Laboratory of Cancer Prevention and Intervention National Ministry of Education) Second Affiliated Hospital Zhejiang University School of Medicine Hangzhou Zhejiang China zju.edu.cn; ^3^ Zhejiang Chinese Medical University Hangzhou China zcmu.edu.cn

**Keywords:** glioma, IDH1 mutation, IDH1 R132H, tumor evolution, tumor recurrence

## Abstract

**Background:**

Isocitrate dehydrogenase 1 (IDH1) mutation is a key molecular marker in gliomas and is generally associated with favorable prognosis. However, its dynamic changes and prognostic significance during tumor recurrence remain incompletely understood.

**Methods:**

We conducted a retrospective analysis of 42 patients with paired primary and recurrent glioma samples. IDH1 mutation status was determined by PCR, and the expression of IDH1 R132H protein was evaluated using a semiquantitative scoring system ranging from negative (−) to strongly positive (+++) based on expression intensity by immunohistochemistry (IHC). Changes in molecular features and WHO grade were analyzed, and their associations with progression‐free survival (PFS), overall survival (OS), and postrecurrence survival (PRS) were evaluated using Cox regression models.

**Results:**

IDH1 mutation status remained largely stable between primary and recurrent tumors, with most patients retaining their original genotype. In contrast, WHO grade progression was observed in 45.2% of cases, indicating ongoing tumor evolution. IDH1 R132H protein expression exhibited dynamic changes, with both upregulation and downregulation detected at recurrence. In primary tumors, IDH1 mutation was independently associated with improved PFS (HR = 0.351, *p* = 0.016), whereas higher IDH1 R132H expression was associated with improved OS (HR = 0.371, *p* = 0.032). In recurrent tumors, IDH1 mutation showed a trend toward inferior PRS, although this association did not reach statistical significance, suggesting that the prognostic relevance of IDH1 alterations may vary during glioma evolution.

**Conclusions:**

Although IDH1 mutation remains largely stable during glioma progression, its prognostic associations may vary between primary and recurrent disease. IDH1 R132H protein expression also shows dynamic changes and may provide complementary prognostic information. These exploratory findings support integrating genetic and protein‐level biomarkers when evaluating glioma progression and warrant validation before informing the clinical application of IDH inhibitors.

**Importance of the Study**

IDH1 mutation is widely regarded as a stable and favorable prognostic marker in gliomas; however, its dynamic role during tumor recurrence remains poorly understood. In this study of 42 paired primary and recurrent glioma samples, we provide preliminary, hypothesis‐generating evidence that the prognostic associations of IDH1 alterations may vary across stages of glioma evolution. Specifically, IDH1 mutation was associated with improved PFS in primary tumors but showed a nonsignificant trend toward worse PRS in recurrent disease. IDH1 R132H protein expression also changed during recurrence and was associated with OS, suggesting that protein‐level assessment may provide complementary information. These exploratory findings warrant validation in larger, molecularly characterized cohorts before they are used for patient stratification or treatment decisions.

## 1. Introduction

Gliomas are the most common primary tumors of the central nervous system and are characterized by marked biological heterogeneity and poor clinical outcomes, particularly in high‐grade disease [1]. Despite advances in surgical resection, radiotherapy, and systemic therapies, tumor recurrence remains almost inevitable and represents a major cause of mortality. Importantly, recurrent gliomas often exhibit substantial molecular and phenotypic evolution, posing significant challenges for prognostic assessment and therapeutic decision‐making [2–4]. Therefore, a deeper understanding of the dynamic changes that occur during glioma progression is essential for improving patient management.

Isocitrate dehydrogenase 1 (IDH1) mutation is one of the most critical molecular alterations in diffuse gliomas. First identified through genomic sequencing studies, IDH1 mutations—most commonly the R132H substitution—are considered early events in gliomagenesis and define biologically distinct tumor subgroups with favorable prognosis and unique metabolic features [5, 6]. Mechanistically, mutant IDH1 produces the oncometabolite D‐2‐hydroxyglutarate (2‐HG), which drives epigenetic reprogramming and tumor development [5]. As a result, IDH1 mutation status has been incorporated into the World Health Organization (WHO) classification and is widely used for diagnosis, prognostication, and therapeutic stratification [1].

In clinical practice, IDH1 R132H protein expression assessed by immunohistochemistry (IHC) is routinely used as a surrogate marker for mutation status [7]. However, emerging evidence suggests that genetic alterations and protein expression may not be entirely concordant, and that protein‐level variation may reflect functional tumor states beyond static genomic classification [8]. In addition, although IDH1 mutation is generally considered stable throughout tumor evolution, recent studies have demonstrated that gliomas undergo continuous genetic and epigenetic remodeling under therapeutic pressure, raising the possibility that the biological and prognostic impact of IDH1 may change during disease progression [9].

Previous studies investigating glioma progression have largely relied on cross‐sectional cohorts or unpaired samples, limiting the ability to capture true longitudinal changes within the same patient. Although histopathological progression—particularly WHO grade escalation—is frequently observed at recurrence, the relationship between molecular stability and phenotypic evolution remains incompletely understood. Furthermore, although IDH1 mutation is widely recognized as a favorable prognostic factor in newly diagnosed gliomas [2], its role in recurrent tumors is less clear, with increasing evidence suggesting that IDH‐mutant gliomas continue to evolve and may acquire more aggressive features over time [10–12].

With the recent development of IDH‐targeted therapies, including ivosidenib and vorasidenib, understanding the temporal stability and functional relevance of IDH1 alterations has become increasingly important for clinical translation [13–15]. Notably, the Phase III INDIGO trial demonstrated significant clinical benefit of vorasidenib in IDH‐mutant low‐grade gliomas, highlighting the therapeutic relevance of this pathway. However, whether IDH1 mutation status and protein expression remain consistent during recurrence—and how these changes influence survival outcomes—remains insufficiently explored.

In this study, we performed a retrospective longitudinal analysis of 42 patients with paired primary and recurrent glioma samples. We systematically evaluated the evolution of IDH1 mutation status, IDH1 R132H protein expression, and WHO tumor grade, and further investigated their associations with progression‐free survival (PFS), overall survival (OS), and postrecurrence survival (PRS). By integrating genetic, protein, and clinical data within a paired‐sample framework, our study aims to provide new insights into the dynamic and stage‐dependent role of IDH1 in glioma progression, and to refine its prognostic and therapeutic implications.

## 2. Methods

### 2.1. Patient and Samples

A total of 42 patients who underwent surgical resection of primary and recurrent gliomas at the Second Affiliated Hospital of Zhejiang University (SAHZU) from January 2008 to December 2015 were included in this study. All samples were subjected to histopathological diagnosis by experienced neuropathologists. This study was approved by the Ethics Committee of SAHZU and conducted in accordance with the Declaration of Helsinki (Approval Number: 046). Written informed consent was obtained from all patients.

### 2.2. PCR

Genomic DNA was extracted from surgically resected glioma specimens using a commercial DNA extraction kit (Sangon Biotech, Shanghai, China) according to the manufacturer′s instructions. The hotspot region of the IDH1 gene was amplified by polymerase chain reaction (PCR) using the following primers: forward 5 ^′^‐GAAGCATAATGTTGGCGTCA‐3 ^′^ and reverse 5 ^′^‐GGGTGTAGATACCAAAAGATAAGAAT‐3 ^′^, generating a 324‐bp fragment.

PCR amplification was performed in a 25 *μ*L reaction system containing template DNA (1 *μ*L), forward and reverse primers (0.5 *μ*L each), dNTPs (10 mM, 0.5 *μ*L), 10× PCR buffer (2.5 *μ*L), Pfu DNA polymerase (0.2 *μ*L; Sangon Biotech), and nuclease‐free water to volume. The PCR cycling conditions were as follows: initial denaturation at 95°C for 3 min; 35 cycles of denaturation at 94°C for 30 s, annealing at 58°C for 30 s, and extension at 72°C for 30–60 s; followed by a final extension at 72°C for 10 min.

PCR products were analyzed by 1.5% agarose gel electrophoresis and subsequently purified using a PCR purification kit (Sangon Biotech). Sanger sequencing was performed on an ABI 3730XL DNA Analyzer (Applied Biosystems, Unite States) to determine IDH1 mutation status. Sequencing data were analyzed using Chromas or SeqMan software.

### 2.3. IHC for IDH1 R132H

Formalin‐fixed, paraffin‐embedded tissue sections from paired primary and recurrent glioma specimens were subjected to IHC staining. After deparaffinization and rehydration, antigen retrieval was performed in EDTA buffer (pH 9.0). Staining was conducted using a mutation‐specific mouse monoclonal antibody against IDH1 R132H (clone H09) (ZSGB‐BIO, China; catalog number: ZM0447). The antibody was applied as a ready‐to‐use solution and incubated for 2 h at room temperature. Immunodetection was performed using a labeled avidin–biotin complex method with 3,3 ^′^‐diaminobenzidine as chromogen (incubation time: 2 min), followed by hematoxylin counterstaining. Positive staining was defined as cytoplasmic immunoreactivity in tumor cells. All immunostained sections were independently assessed by two experienced pathologists blinded to clinical information. The semiquantitative scoring system divides IHC results into four levels based on expression intensity: −, +, ++, and +++ [8].

### 2.4. Statistical Analysis

Statistical analyses were performed using GraphPad Prism (Version 10) and R software. Survival outcomes, including OS, PFS, and PRS, were estimated using the Kaplan–Meier method and compared using the log‐rank test. Given the limited sample size and potential risk of model overfitting, multivariable Cox regression models were restricted to clinically relevant variables to maintain an adequate events‐per‐variable ratio. Firth penalized Cox regression was subsequently performed to reduce small‐sample bias and improve estimation stability. Internal validation was conducted using 1000 bootstrap resampling iterations, and median hazard ratios with bootstrap confidence intervals were calculated to assess model robustness. All statistical tests were two‐sided, and a *p* value < 0.05 was considered statistically significant.

## 3. Results

### 3.1. Clinicopathological Features of Paired Primary and Recurrent Gliomas Stratified by IDH1 Status

A total of 42 patients with paired primary and recurrent tumor samples were included in the analysis (Table 1). At the time of primary diagnosis, 34 patients (81.0%) were classified as IDH1‐WT, whereas 8 patients (19.0%) harbored IDH1 mutations. At recurrence, 32 patients (76.2%) were IDH1‐WT and 10 patients (23.8%) were IDH1‐mutant.

**Table 1:** Characteristics.

		Primary		Recurrence	
		IDH wt	IDH mutant	IDH wt	IDH mutant
Patients		34 (81.0%)	8 (19.0%)	32 (76.2%)	10 (23.8%)

Age		41.56 (11–61)	50.13 (33–60)	43.34 (11–61)	42.7 (22–60)

Sex	Male	19 (45.2%)	8 (19.0%)	21 (50.0%)	6 (14.3%)
	Female	15 (35.7%)	0 (0.0%)	11 (26.2%)	4 (9.5%)

Residual	Yes	17 (40.5%)	4 (9.5%)	14 (33.3%)	7 (16.7%)
	No	17 (40.5%)	4 (9.5%)	18 (42.9%)	3 (7.1%)

Adjuvant therapy	Radiotherapy alone	4 (9.5%)	2 (4.8%)	4 (9.5%)	2 (4.8%)
	Chemotherapy alone	1 (2.4%)	0 (0.0%)	1 (2.4%)	0 (0.0%)
	Chemoradiotherapy	12 (28.6%)	3 (7.1%)	15 (35.7%)	0 (0.0%)
	No adjuvant therapy	17 (40.5%)	3 (7.1%)	12 (28.6%)	8 (19.0%)

IHC of IDH1	No expression (−)	23 (54.8%)	3 (7.1%)	22 (52.4%)	4 (9.5%)
	Low expression (+)	4 (9.5%)	3 (7.1%)	5 (11.9%)	0 (0.0%)
	Moderate expression (++)	6 (14.3%)	1 (2.4%)	3 (7.1%)	3 (7.1%)
	High expression (+++)	1 (2.4%)	1 (2.4%)	2 (4.8%)	3 (7.1%)

WHO Grades	1	1 (2.4%)	1 (2.4%)	1 (2.4%)	0 (0.0%)
	2	8 (19.0%)	2 (4.8%)	5 (11.9%)	2 (4.8%)
	3	11 (26.2%)	1 (2.4%)	8 (19.0%)	3 (7.1%)
	4	14 (33.3%)	4 (9.5%)	18 (42.9%)	5 (11.9%)

Regarding sex distribution, among the IDH1‐WT group at primary diagnosis, 19 patients (45.2%) were male and 15 (35.7%) were female, whereas all 8 IDH1‐mutant cases were male. At recurrence, 21 males (50.0%) and 11 females (26.2%) were classified as IDH1‐WT, whereas 6 males (14.3%) and 4 females (9.5%) were IDH1‐mutant.

The median age at primary diagnosis was 41.56 years (range, 11–61) in the IDH1‐WT group and 50.13 years (range, 33–60) in the IDH1‐mutant group. At recurrence, the median age was 43.34 years (range, 11–61) and 42.7 years (range, 22–60) for IDH1‐WT and IDH1‐mutant patients, respectively.

Through IHC staining of IDH1 R132H at primary diagnosis, 23 patients (54.8%) in the IDH1‐WT group and 3 patients (7.1%) in the IDH1‐mutant group showed no expression. Low expression was observed in 4 (9.5%) IDH1‐WT and 3 (7.1%) IDH1‐mutant tumors. Moderate expression occurred in 6 (14.3%) and 1 (2.4%), whereas high expression was detected in 1 (2.4%) patient in each group. At recurrence, no expression was observed in 22 patients (52.4%) with IDH1‐WT and 4 patients (9.5%) with IDH‐mutant tumors, whereas moderate and high expression were observed in 3 (7.1%) and 3 (7.1%) IDH‐mutant tumors, respectively.

With respect to tumor grade, at primary diagnosis, WHO Grade 4 tumors accounted for the largest proportion in the IDH1‐WT group (14 patients, 33.3%), followed by grade 3 (11 patients, 26.2%) and Grade 2 (8 patients, 19.0%). In contrast, among IDH1‐mutant tumors, 4 patients (9.5%) were grade 4, whereas Grades 1, 2, and 3 each accounted for 1–2 patients. At recurrence, 18 patients (42.9%) with IDH1‐WT tumors were classified as WHO Grade 4, whereas 5 patients (11.9%) in the IDH‐mutant group were Grade 4.

### 3.2. Evolution of IDH1 Status, Protein Expression, and Tumor Grade During Recurrence

To further explore molecular and pathological changes during tumor progression, we compared IDH1 genetic status, IDH1 R132H protein expression, and WHO grades between primary and recurrent tumors (Table 2).

**Table 2:** Differences between primary and recurrent lesions.

Characteristics	Patients no.(%)
IDH1 gene sequencing(primary→recurrence)	
WT → WT	28 (66.7%)
R132H → R132H	3 (7.1%)
WT → R132H	4 (9.5%)
R132H → WT	3 (7.1%)
c.98A>G → WT	1 (2.4%)
R132S, R132G → R132G	1 (2.4%)
WT → c.64T>C	1 (2.4%)
WT → c.67G>A	1 (2.4%)
IDH1 expression by IHC(primary→recurrence)	
Same	26 (61.9%)
Upgrade	10 (23.8%)
Downgrade	6 (14.2%)
WHO grades(primary→recurrence)	
Same	22 (52.4%)
Upgrade	19 (45.2%)
Downgrade	1 (2.4%)

Regarding IDH1 gene status, the majority of patients showed stable IDH1 status between the primary and recurrent tumors. Specifically, 28 patients (66.7%) remained IDH1‐WT (WT → WT), whereas 3 patients (7.1%) retained the R132H mutation (R132H → R132H). Notably, 4 patients (9.5%) exhibited a change from WT to R132H mutation (WT → R132H) at recurrence, whereas 3 patients (7.1%) showed the opposite change (R132H → WT). In addition, several rare mutation pattern changes were observed, including c.98A>G → WT (1 patient, 2.4%), R132S and R132G → R132G (1 patient, 2.4%), WT → c.64T>C (1 patient, 2.4%), and WT → c.67G>A (1 patient, 2.4%).

Analysis of IDH1 R132H expression by IHC revealed that 26 patients (61.9%) showed no change in expression levels between primary and recurrent tumors. However, 10 patients (23.8%) demonstrated increased expression (upgrade) at recurrence, whereas 6 patients (14.2%) exhibited decreased expression (downgrade).

With respect to tumor grading, 22 patients (52.4%) maintained the same WHO grade at recurrence. Tumor grade progression was observed in 19 patients (45.2%), whereas only 1 patient (2.4%) showed grade reduction at recurrence (Figure 1B).

**Figure 1:**
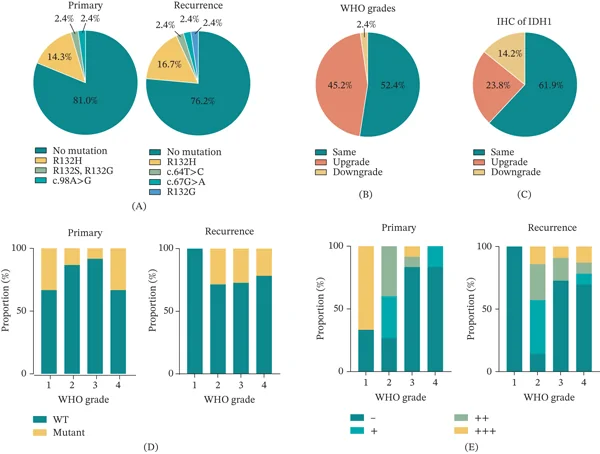
Comparison of IDH1 mutation status, WHO grade, and IHC expression between primary and recurrent gliomas. (A) Distribution of IDH1 mutation status in primary (left) and recurrent (right) tumor samples. (B) Changes in WHO grade between primary and recurrent tumors. (C) Changes in IDH1 R132H IHC expression levels between primary and recurrent tumors. (D) Proportion of IDH1‐WT and mutant tumors stratified by WHO grade in primary (left) and recurrent (right) gliomas. (E) Distribution of IDH1 R132H expression levels (−, +, ++, +++) across WHO grades in primary (left) and recurrent (right) gliomas.

Overall, these findings indicated that although most patients maintained stable IDH1 genetic status and protein expression, a substantial proportion exhibited WHO grade progression during tumor recurrence.

### 3.3. Molecular Stability With Phenotypic Progression in Recurrent Gliomas

IDH1 status revealed that the majority of tumors were WT in both primary and recurrent gliomas. The most common mutation subtype was R132H, accounting for 14.3% of primary tumors and 16.7% of recurrent tumors, whereas other rare mutation types were detected at low frequencies. Overall, the distribution of IDH1 mutation status showed minimal differences between primary and recurrent samples, indicating relative molecular stability during tumor progression (Figure 1A). In contrast, histopathological features exhibited changes that are more dynamic. WHO grade analysis demonstrated that a substantial proportion of tumors underwent grade progression at recurrence, with fewer cases remaining stable or showing downgrading (Figure 1B). Consistently, immunohistochemical analysis revealed that IDH1 R132H expression levels were altered in a subset of patients, with a tendency toward increased expression in recurrent tumors (Figure 1C). To further explore the relationship between IDH1 status and tumor grade, we analyzed the distribution of IDH1 mutation across WHO grades. IDH1 mutations were more frequently observed in lower‐grade tumors, whereas high‐grade tumors were predominantly WT in both primary and recurrent settings (Figure 1D). Similarly, stratification by IHC demonstrated that stronger IDH1 R132H expression was more common in lower‐grade tumors, while higher‐grade tumors tended to exhibit reduced expression levels (Figure 1E).

In primary tumors, among IDH1‐WT tumors, 67.6% showed negative staining, whereas IDH1‐mutant tumors showed a more even distribution across expression levels, including higher proportions of positive staining (Figure 2A). In recurrent tumors, a similar pattern was observed: WT cases remained largely negative (68.8%), whereas mutant tumors exhibited increased proportions of moderate to strong expression (Figure 2B). Notably, high (+++) expression was more frequent in IDH1‐mutant tumors compared with WT tumors in both primary and recurrent samples.

**Figure 2:**
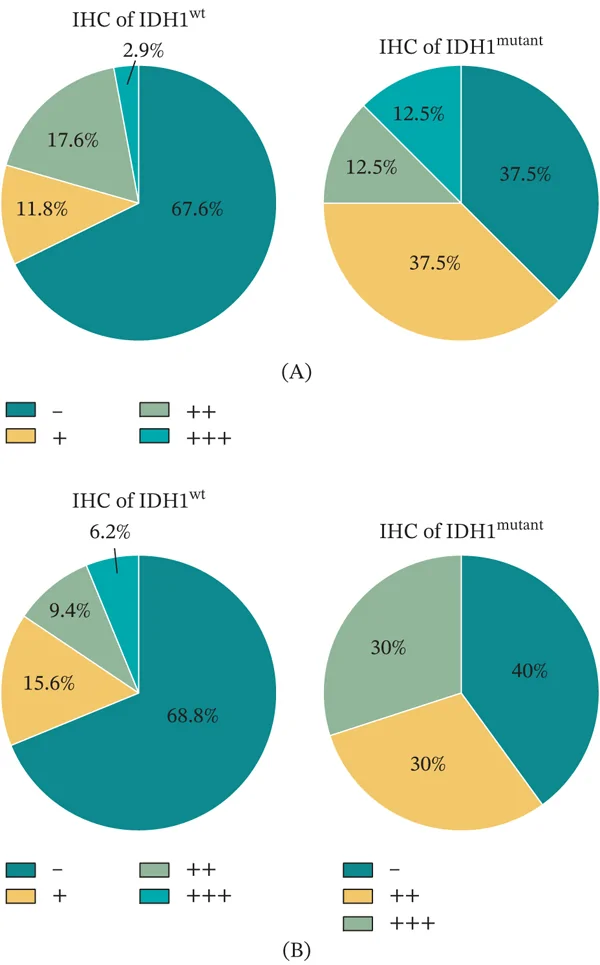
Distribution of IDH1 R132H expression intensity in primary and recurrent gliomas stratified by IDH1 mutation status. (A) Primary tumor samples. Left: IDH1‐WT cases; right: IDH1‐mutant cases. (B) Recurrent tumor samples. Left: IDH1‐WT cases; right: IDH1‐mutant cases. IHC staining intensity was categorized as negative (−), weak (+), moderate (++), and strong (+++).

One representative case showed discordant IDH1 status, changing from IDH1‐WT at primary diagnosis to IDH1‐mutant at recurrence, accompanied by increased IDH1 R132H protein expression (Figure 3A,B). In IDH1 sequencing, a change in the 132nd nucleotide site was found, from G to A (Figure 3C).

**Figure 3:**
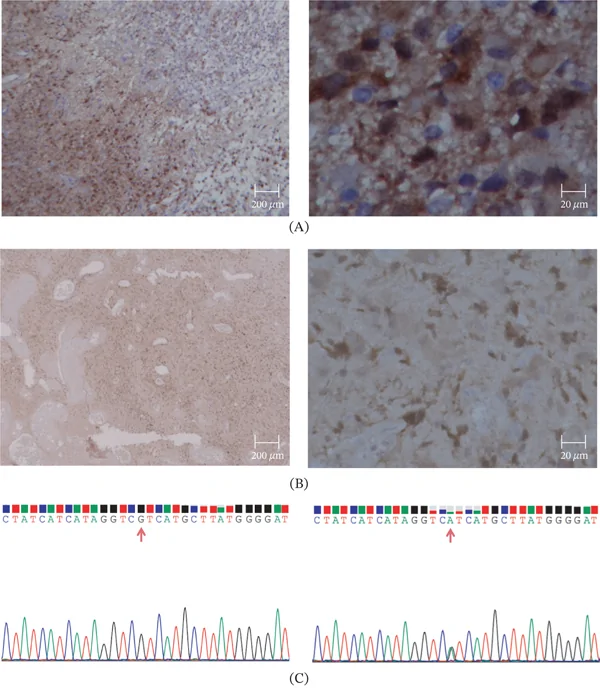
Immunohistochemical analysis of IDH1 R132H expression in paired primary and recurrent glioma samples. (A) Representative immunohistochemical staining of IDH1 in primary glioma surgical specimens. (B) Representative immunohistochemical staining of IDH1 in recurrent glioma surgical specimens. Left: low‐magnification images (×5). Right: high‐magnification images (×40). Scale bars, 200 *μ*m. (C) Changes in base sites in IDH1 sequencing.

### 3.4. Prognostic Significance of Molecular and Clinical Variables

Firth penalized Cox regression analysis for PFS is summarized in Table S1. Compared with WHO Grade 1–3 tumors, Grade 4 tumors had a significantly higher risk of progression (HR = 4.994, 95% CI: 1.950–12.899, *p* = 0.001). IDH1 mutation was significantly associated with improved PFS after Firth penalization (HR = 0.351, 95% CI: 0.135–0.828, *p* = 0.016). Higher IDH1 R132H expression by IHC showed a favorable association with PFS, but the association was not statistically significant (Figure 4A).

**Figure 4:**
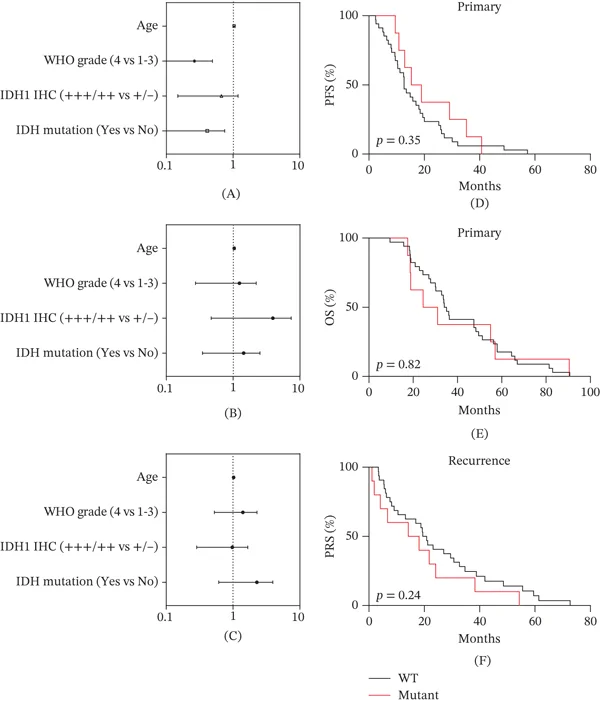
Firth penalized Cox regression and survival analyses of prognostic factors for PFS, OS, and PRS. (A) Firth penalized Cox regression analysis of clinical and molecular factors of primary glioma associated with PFS of patients. (B) Firth penalized Cox regression analysis of clinical and molecular factors of primary glioma associated with OS of patients. (C) Firth penalized Cox regression analysis of clinical and molecular factors of recurrence glioma associated with PRS of patients. (D–F) Kaplan–Meier survival curves comparing patients with IDH1‐WT and mutant tumors. (D) PFS in primary glioma patients. (E) OS in primary glioma patients. (F) PRS in recurrent glioma patients.

Firth penalized Cox regression analysis for OS is presented in Table S2. Older age was significantly associated with worse OS (HR = 1.038, 95% CI: 1.006–1.073, *p* = 0.017). Higher IDH1 R132H expression (+++/++ vs +/−) was associated with improved OS (HR = 0.371, 95% CI: 0.139–0.920, *p* = 0.032). Higher WHO grade and IDH1 mutation showed directional associations with poorer OS, but neither association was statistically significant (Figure 4B).

Firth penalized Cox regression analysis for PRS is presented in Table S3. In the recurrence model, older age, higher WHO grade, and IDH1 mutation showed directional associations with shorter PRS, but none reached statistical significance. Higher IDH1 R132H expression showed a directional association with longer PRS, but this association was also not statistically significant (Figure 4C).

Neither PFS (17.15 m vs. 12.7 m, *p* = 0.35) nor OS (27.75 m vs. 34.5 m, *p* = 0.82) differed significantly between primary tumors with IDH1‐mutant and WT. Similarly, PRS was not significantly associated with recurrence tumors with different IDH1 mutation status (16.5 m vs. 20.1 m, *p* = 0.24). Although IDH1‐mutant tumors exhibited a trend toward prolonged PFS but shorter PRS compared with WT tumors, none of these differences reached statistical significance. These findings suggest that IDH1 mutation status has limited prognostic value in survival analyses (Figure 4D–F).

Bootstrap validation using 1000 resampling iterations demonstrated consistent HR directions across PFS, OS, and PRS models (Tables S4–6), supporting the stability of the penalized regression estimates.

Overall, these results demonstrate that tumor grade and IDH1 mutation status are the primary determinants of disease progression, as reflected by their significant associations with PFS. In contrast, OS was more strongly associated with clinical and protein‐level factors, including age and IDH1 R132H expression. In recurrent tumors, no molecular factors reached statistical significance for PRS, although directional trends were observed, highlighting a dynamic and context‐dependent shift in prognostic factors over the disease course. Collectively, these findings underscore the complex interplay between tumor biology and treatment‐related factors in shaping survival outcomes in glioma patients.

## 4. Discussion

In this study, we performed a longitudinal analysis of paired primary and recurrent glioma samples from 42 patients to investigate the dynamic changes in IDH1 genetic status, IDH1 R132H protein expression, and tumor grading, as well as their prognostic significance. Our findings reveal a stage‐dependent and context‐dependent association between IDH1 alterations and clinical outcomes, characterized by genetic stability but distinct prognostic implications across different disease stages.

Although IDH1 mutation status remained stable in most paired samples, seven patients showed WT ↔ R132H status changes between primary and recurrent tumors; their clinical and pathological characteristics are summarized in Table S7. All paired specimens contained more than 80% tumor cells on pathological review, although retrospective quantitative tumor‐purity estimates were unavailable. Tumor phenotypes nevertheless showed substantial plasticity, and a considerable proportion of patients experienced WHO grade progression at recurrence. Because many tumors were already Grade 4 at baseline, grade stability alone may underestimate biological evolution 9. IDH1 mutations are generally considered early truncal events that are maintained throughout gliomagenesis, but several factors may explain the observed discrepancies. Intratumoral heterogeneity and sampling bias may result in spatially distinct subclones with different proportions of IDH1‐altered cells. Technical factors, including DNA quality and assay sensitivity, may also influence mutation detection. Although less likely, true clonal evolution under therapeutic pressure cannot be excluded. These cases should therefore be interpreted as discordant IDH1 status, rather than definitive evidence of mutation acquisition or loss.

At the protein level, the expression of IDH1 R132H showed dynamic changes during recurrence, with upregulation and downregulation observed. It is worth noting that compared with IDH1‐WT tumors, IDH1‐mutant tumors have a wider distribution of expression intensity. These findings suggest that IDH1 mutations and IDH1 R132H protein expression represent different biological layers, as protein expression may reflect downstream functional activity and tumor status, rather than just genetic status. This may be because of IDH1 mutations leading to the production of tumor metabolite 2‐HG, which induces epigenetic reprogramming and alters the behavior of tumor cells. Further studies using larger paired cohorts are warranted to elucidate the underlying mechanisms. The association between higher IDH1 R132H expression and improved OS suggests that protein‐level assessment may capture additional biological information beyond mutation status, although the underlying mechanisms remain unclear.

A key finding of this study is that the prognostic association of IDH1 mutation differed across disease stages. In primary tumors, IDH1 mutation was significantly associated with improved PFS after adjustment for available clinical variables, which is consistent with previous studies [2]. However, IDH‐mutant tumors are not “inert benign lesion” but rather continuously evolving invasive tumor [10]. In recurrent tumors, IDH1 mutation showed a trend toward inferior PRS, although this association did not reach statistical significance. These findings suggest that the clinical implication of IDH1 mutation may change during tumor evolution and requires validation in larger cohorts.

Several biological mechanisms may contribute to the stage‐dependent prognostic pattern observed in our study. First, recurrent IDH‐mutant glioma frequently acquire additional genetic and epigenetic alterations such as CDKN2A delta, aneuploidy, hypermutation, and transition from G‐CIMP^high^ to G‐CIMP^low^ that may reduce their dependence on the canonical IDH‐driven pathway [4, 11, 12]. Previous studies found that IDH‐mutant gliomas initially have high levels of whole genome DNA methylation, but gradually lose methylation and approach a state closer to IDH‐WT after recurrence, especially after radiotherapy. Changes from GCIMP^high^ to GCIMP^low^ may occur in recurrent IDH‐mutant tumors, indicating a poorer prognosis [4]. Second, therapeutic pressure from prior treatments may lead to the selection of more aggressive subclones within IDH1‐mutant tumors [9]. Third, tumor–microenvironment interactions, including metabolic and immune remodeling, may significantly alter tumor behavior at recurrence [16, 17]. Recent studies have shown that epigenetic and transcriptional reprogramming can reshape tumor progression independently of initial driver mutations, further supporting this hypothesis [11].

Another important finding is that IDH1 R132H protein expression was associated with improved OS in primary tumors, whereas IDH1 mutation status alone was not significantly associated with OS. This suggests that protein‐level alterations may capture complementary biological information beyond genetic status. We found that not all tumors with IDH1 R132H mutation detected by PCR were IHC positive, nor were all IDH1‐WT tumors IHC negative. Therefore, our results support the concept that the biological impact of IDH1 depends not only on mutation status but also on its functional expression level. The discrepancy between IDH1 mutation and protein expression further highlights the importance of multilevel molecular assessment. Although mutation status provides a stable classification framework, protein expression may capture real‐time tumor activity and aggressiveness. This distinction becomes particularly relevant in recurrent tumors, where tumor biology is shaped by both intrinsic evolution and external therapeutic pressures.

The relative stability of IDH1 mutation status observed in our cohort may have therapeutic implications. Most paired tumors retained their status, whereas a small subset showed discordant results at recurrence; these findings should not be interpreted as definitive mutation acquisition or loss. The Phase 3 INDIGO trial has confirmed the efficacy of vorasidenib in patients with low‐grade IDH‐mutant glioma [10, 13]. Therefore, it is necessary to validate IDH1 inhibitors in different grades of gliomas to expand the patient population that can benefit from these therapies.

However, the dynamic changes observed in IDH1 R132H protein expression by IHC indicated that, despite genetic stability, functional activity at the protein level may vary over time. This raises the possibility that therapeutic response to IDH1 inhibitors could be influenced not only by mutation status but also by expression levels and tumor heterogeneity. In addition, the differences observed in survival data and the expression of IDH1 at the gene and protein levels in our study suggest that IDH1 has a more complex impact on tumor progression, targeted therapy efficacy, and patient survival than expected. Although IDH1 mutation is generally considered a favorable prognostic marker in glioma, its prognostic significance is not uniform across clinical contexts. In previous studies, similar to our findings, IDH1 mutation in gliomas was significantly associated with improved PFS after adjustment for available clinical variables, whereas there was no statistically significant difference in their effect on OS [2, 18].

Several limitations should be acknowledged. This was a small, single‐center retrospective study, and the multivariable survival analyses were susceptible to overfitting. We reduced model complexity, applied Firth penalized Cox regression, and performed bootstrap internal validation; nevertheless, the findings require confirmation in larger independent paired cohorts. Treatment regimens were heterogeneous, and detailed radiotherapy and chemotherapy parameters were not consistently available, leaving potential residual treatment‐related confounding. Comprehensive molecular profiling was also unavailable. Specifically, O6‐methylguanine‐DNA methyltransferase (MGMT) promoter methylation, 1p/19q codeletion, alpha‐thalassemia/mental retardation syndrome X‐linked (ATRX) loss, and telomerase reverse transcriptase (TERT) promoter status were not systematically assessed. Because these markers are biologically linked to IDH1 status and may influence treatment response and survival, their absence further limits causal or independent prognostic interpretation. The observed associations should therefore be considered exploratory and hypothesis‐generating.

In conclusion, IDH1 mutation status remained largely stable during glioma recurrence, whereas tumor grade and protein expression changed in a subset of patients. The observed survival associations were stage‐dependent and context‐dependent but exploratory. Larger, molecularly characterized paired cohorts are needed to validate these findings and determine their relevance to molecular stratification and IDH‐targeted therapy.

## Author Contributions

S.G. and B.L. contributed to the data collection and manuscript writing. W.Y. and Q.W. contributed to the review, discussion, and language editing. W.Y. contributed to the sample collection and testing. Y.W., S.G., and B.L. contributed equally to this work.

## Funding

This study was supported by the National Natural Science Foundation of China, 10.13039/501100001809, 82473234.

## Disclosure

All the authors have read and approved the final manuscript and provided valuable critical discussion. All scientific interpretations, data analyses, and conclusions were independently performed and verified by the authors.

## Conflicts of Interest

The authors declare no conflicts of interest.

## Supporting Information

Additional supporting information can be found online in the Supporting Information section.

## Supporting information


**Supporting Information 1** Table S1: Multivariable Firth penalized Cox regression analysis of progression‐free survival.

**Supporting Information 2** Table S2: Multivariable Firth penalized Cox regression analysis of overall survival.

**Supporting Information 3** Table S3: Multivariable Firth penalized Cox regression analysis of postrecurrence survival.

**Supporting Information 4** Table S4: Bootstrap validation of the progression‐free survival model.

**Supporting Information 5** Table S5: Bootstrap validation of the overall survival model.

**Supporting Information 6** Table S6: Bootstrap validation of the postrecurrence survival model.

**Supporting Information 7** Table S7: Clinical and pathological characteristics of seven patients with IDH1 WT ↔ R132H status changes between primary and recurrent gliomas.

## Data Availability

The datasets used and analyzed during the current study are available from the corresponding author on reasonable request.
