# Supplementary material for: Dynamics of IDH1 Mutation and Protein Expression Between Primary and Recurrent Gliomas and Their Prognostic Significance
Source: Hum Mutat. 2026 Oct 27;2026:7543241. doi: 10.1155/humu/7543241 (PMC13711253; doi:10.1155/humu/7543241)
Supplement: Supplementary file 1 [file HUMU-2026-7543241-s002.docx]

**Supplementary Table 3. Multivariable Firth penalized Cox regression analyses of PRS.**

| **Variable** | **HR, 95% CI** | **P value** |
| --- | --- | --- |
| Age | 1.020 (0.997–1.045) | 0.091 |
| WHO grade (4 vs 1-3) | 1.205 (0.633–2.314) | 0.569 |
| IDH1 R132H IHC (+++/++ vs +/−) | 0.839 (0.381–1.728) | 0.643 |
| IDH1 mutation (Yes vs No) | 1.943 (0.858–4.127) | 0.108 |

Firth penalized Cox regression models were fitted using penalized maximum likelihood estimation. HRs and 95% CIs were estimated using profile likelihood methods.
