# Supplementary material for: Dynamics of IDH1 Mutation and Protein Expression Between Primary and Recurrent Gliomas and Their Prognostic Significance
Source: Hum Mutat. 2026 Oct 27;2026:7543241. doi: 10.1155/humu/7543241 (PMC13711253; doi:10.1155/humu/7543241)
Supplement: Supplementary file 2 [file HUMU-2026-7543241-s003.docx]

**Supplementary Table 2. Multivariable Firth penalized Cox regression analyses of OS.**

| **Variable** | **HR, 95% CI** | **P value** |
| --- | --- | --- |
| Age | **1.038 (1.006–1.073)** | **0.017** |
| WHO grade (4 vs 1-3) | 1.063 (0.455–2.379) | 0.885 |
| IDH1 R132H IHC (+++/++ vs +/−) | **0.371 (0.139–0.920)** | **0.032** |
| IDH1 mutation (Yes vs No) | 1.243 (0.520–2.688) | 0.606 |

Firth penalized Cox regression models were fitted using penalized maximum likelihood estimation. HRs and 95% CIs were estimated using profile likelihood methods.
