# Supplementary material for: Dynamics of IDH1 Mutation and Protein Expression Between Primary and Recurrent Gliomas and Their Prognostic Significance
Source: Hum Mutat. 2026 Oct 27;2026:7543241. doi: 10.1155/humu/7543241 (PMC13711253; doi:10.1155/humu/7543241)
Supplement: Supplementary file 3 [file HUMU-2026-7543241-s004.docx]

**Supplementary Table 1. Multivariable Firth penalized Cox regression analyses of PFS.**

| **Variable** | **HR, 95% CI** | **P value** |
| --- | --- | --- |
| Age | 1.031 (0.993–1.076) | 0.114 |
| WHO grade (4 vs 1–3) | **4.994 (1.950–12.899)** | **0.001** |
| IDH1 R132H IHC (+++/++ vs +/−) | 0.571 (0.235–1.266) | 0.172 |
| IDH1 mutation (Yes vs No) | **0.351 (0.135–0.828)** | **0.016** |

Firth penalized Cox regression models were fitted using penalized maximum likelihood estimation. HRs and 95% CIs were estimated using profile likelihood methods.
