# Supplementary material for: Dynamics of IDH1 Mutation and Protein Expression Between Primary and Recurrent Gliomas and Their Prognostic Significance
Source: Hum Mutat. 2026 Oct 27;2026:7543241. doi: 10.1155/humu/7543241 (PMC13711253; doi:10.1155/humu/7543241)
Supplement: Supplementary file 5 [file HUMU-2026-7543241-s006.docx]

**Supplementary Table 5. Bootstrap validation of the Firth penalized Cox regression model for OS**

| **Variable** | **Median HR** | **Bootstrap 95% CI** |
| --- | --- | --- |
| Age | 1.042 | 1.016–1.090 |
| WHO grade (4 vs 1-3) | 1.035 | 0.299–4.140 |
| IDH1 R132H IHC (+++/++ vs +/−) | 0.341 | 0.083–0.869 |
| IDH1 mutation (Yes vs No) | 1.157 | 0.434–3.570 |

Bootstrap validation was performed using 1,000 resampled datasets. Median HRs and 95% bootstrap confidence intervals were calculated to assess model stability.
