# Supplementary material for: Dynamics of IDH1 Mutation and Protein Expression Between Primary and Recurrent Gliomas and Their Prognostic Significance
Source: Hum Mutat. 2026 Oct 27;2026:7543241. doi: 10.1155/humu/7543241 (PMC13711253; doi:10.1155/humu/7543241)
Supplement: Supplementary file 6 [file HUMU-2026-7543241-s007.docx]

**Supplementary Table 4. Bootstrap validation of the Firth penalized Cox regression model for PFS**

| **Variable** | **Median HR** | **Bootstrap 95% CI** |
| --- | --- | --- |
| Age | 1.029 | 0.984–1.111 |
| WHO grade (4 vs 1-3) | 5.123 | 2.410–17.371 |
| IDH1 R132H IHC (+++/++ vs +/−) | 0.558 | 0.181–1.815 |
| IDH1 mutation (Yes vs No) | 0.318 | 0.107–0.766 |

Bootstrap validation was performed using 1,000 resampled datasets. Median HRs and 95% bootstrap confidence intervals were calculated to assess model stability.
